# Application of 3D Simulation Software in Chemotherapy and Hepatoblastoma Surgery in Children

**DOI:** 10.3389/fsurg.2022.908381

**Published:** 2022-06-01

**Authors:** Jie Liu, Wenli Xiu, Guangqi Duan, Qian Dong

**Affiliations:** ^1^ Department of Pediatric Surgery, Yijishan Hospital of Wannan Medical College, Wannan Medical College, Wuhu, China; ^2^ Department of Pediatric Surgery, Affiliated Hospital of Qingdao University, Qingdao University, Qingdao, China

**Keywords:** computer-assisted surgery, hepatoblastoma, chemotherapy, three-dimensional, children

## Abstract

**Purpose:**

This study aims to explore the clinical value of a computer-assisted surgery system (Hisense CAS) in hepatoblastoma (HB) surgery in children after neoadjuvant chemotherapy.

**Patients and Methods:**

The clinical medical records of children with HB treated after neoadjuvant chemotherapy at the Affiliated Hospital of Qingdao University from January 2016 to January 2019 were analyzed retrospectively.

**Results:**

A total of 21 children were enrolled in this study, including 13 boys and 8 girls. All cases successfully underwent three-dimensional (3D) reconstruction of the liver and tumor using Hisense CAS, simulated hepatectomy, and hepatectomy according to the preoperative operation plan. There were twelve cases of right hemihepatectomy, four cases of right trefoil hepatectomy, one case of left lobe hepatectomy, and three cases of middle lobe hepatectomy, and one case of V and VI segment hepatectomy. All children recovered well after the operation. The follow-up ranged from 5 months to 3 years. One child died of systemic metastasis 8 months after the operation. One child received one course of chemotherapy after the operation. Due to the serious reaction to the chemotherapy, the family refused further treatment and follow-up. The remaining 19 children had no complications or recurrence.

**Conclusion:**

Hisense CAS can clearly and intuitively display the position and shape of the HB before and after chemotherapy and its relationship with the intrahepatic pipeline system and accurately evaluate the changes in tumor volume and the distance between important blood vessels, which is conducive to the operator selecting the best operation opportunity, timely formulating the best operation plan and implementing individualized and accurate liver tumor resection.

## Introduction

Hepatoblastoma (HB) is the most common malignant tumor of the liver in children, accounting for approximately 80% of the total number of liver tumors in childhood (1–3). The incidence of HB in children is third among cancers, following neuroblastoma and Wilms’ tumor (4). The onset age of HB generally ranges from 6 months to 3 years. It is rare after 5 years of age. The incidence of the disease is higher in boys than in girls (5, 6). Although complete resection of the tumors is the main method for treating HB, many children have an advanced tumor stage at the time of diagnosis and the tumor is unresectable (7–9).

After the 1980s, due to the use of effective neoadjuvant drug treatment, especially the application of chemotherapy based on platinum drugs, a large number of patients who initially could not be operated on have achieved complete tumor resection and the prognosis has greatly improved, with a 5-year survival rate reaching 75% or even higher (10, 11). Some clinicians believe that if HB can undergo surgical treatment after two cycles of chemotherapy, early resection should be attempted. If the tumor cannot be surgically removed after four cycles, liver transplantation is recommended instead of further chemotherapy, and they believe that HB tumors enter a stagnation period after two cycles, and the tumor may develop drug resistance with additional chemotherapy (12–14). Therefore, it is particularly important for patients with HB to timely evaluate the liver imaging data before and after chemotherapy, accurately measure and observe the relationship between the tumor and surrounding blood vessels and tissues, and select the best operation time.

When using traditional B-ultrasound, CT, and other 2D imaging data, it is difficult to accurately judge the scope of tumor invasion, the blood supply and the adjacent relationship with surrounding blood vessels, and changes in the shape of intrahepatic pipelines before and after HB chemotherapy, and it is also difficult to accurately judge whether the tumor can be completely removed, the scope of resection, and the method of resection (15, 16).

In this study, the two-dimensional (2D) CT data of patients with HB before and after neoadjuvant chemotherapy were imaged by a Hisense computer-assisted surgical (Hisense CAS) system. This system is used for virtual hepatectomy before the operation and for real-time navigation during the operation. The goal of this study was to evaluate the role and value of Hisense CAS in the operation of patients with HB undergoing chemotherapy.

## Materials and Methods

### Patients and Clinical Parameters

The clinical data of the children with HB who received preoperative neoadjuvant chemotherapy and then underwent surgery at the Affiliated Hospital of Qingdao University from January 2016 to January 2019 were analyzed retrospectively. All children underwent an enhanced CT scan of the upper abdomen, alpha-fetoprotein (AFP) examination, and fine needle biopsy before the operation. Each patients treatment plan was formulated by a multidisciplinary team comprised of pediatric surgeons, radiologists, and child oncologists. The study excluded patients with other malignancies, poor compliance, and no postoperative follow-up. This study was approved by the ethics committee of the Affiliated Hospital of Qingdao University (QYFY-WZLL-25776). The parents of patients with HB were informed, and they signed informed consent before the operation.

### Imaging Evaluation

All patients underwent abdominal enhanced CT examination and three-dimensional (3D) reconstruction imaging by Hisense CAS at the time of admission for diagnosis and puncture biopsy of the HB under the guidance of B-ultrasound to further clarify the diagnosis and confirm the histological type. According to the Childrens Oncology Group (COG) surgery guidelines (AHEP-0731) (14), for patients who are recommended to undergo standard-risk surgical tumor resection, 2–4 cycles of the C5 V chemotherapy regimen are used. In the C5 V chemotherapy regimen, cisplatin (100 mg/m^2^/day) should be continuously administered intravenously for at least 6 h protected from light on the first day of the start of chemotherapy, 5-fluorouracil (600 mg/m^2^/day) should be given by IV push (not over 4 h) on the second day, and vincristine (1.5 mg/m^2^/day) should be administered on the second, ninth, and sixteenth days. Three weeks later, if neutrophils ≥1.0 × 109/L, platelets (PLTs) ≥ 100 × 109/L, and liver and kidney functions are normal, the next course of chemotherapy is administered. In high-risk patients, tumor biopsy is usually performed first, followed by 2–4 cycles of chemotherapy including C5V and doxorubicin (D; 30 mg/m^2^/day on days 1 and 2) after pathological diagnosis. The neoadjuvant chemotherapy indications and chemotherapy regimens were described in detail in our previous studies (10). After two cycles of chemotherapy, enhanced CT examination and 3D reconstruction by the computer-assisted surgery system were performed again to evaluate the resectability of the tumor. If it was still unresectable, one or two cycles of chemotherapy and imaging evaluation were applied. The evaluation of patients after chemotherapy mainly includes the following: (1) Patients evaluated as POST-TEXT I and II can be treated surgically. Patients who are evaluated as POST-TEXT III and have no important vascular (portal vein or inferior vena cava) invasion can be treated with lobectomy or segmental hepatectomy. (2) Patients evaluated as POST-TEXT III with important vascular involvement or POST-TEXT IV will receive liver transplantation.

### Hisense CAS Was Used for 3D Reconstruction of Tumors

The 3D reconstruction steps of the Hisense CAS system have been described in detail in our teams previous paper (17). The specific reconstruction method is roughly divided into the following three parts:

(i) 3D reconstruction of the liver: Import the DICOM format file of 0.625 mm thin-layer enhanced CT scanning into the Hisense CAS system, adjust the window width and window level, identify the liver edge through artificial intelligence and traditional image segmentation algorithms, and segment it accurately. The results are compared and verified on the original 2D image data through mask addition and the provided interactive tools, which can locally fine-tune the segmentation results, reconstruct the liver, and calculate the liver volume.
(ii) 3D reconstruction of HB: Select the segmentation seed points at the tumor location, draw a closed curve in the tumor area in the cross section, mark the tumor, and segment the tumor in different cross sections of the coronal plane and sagittal plane, and the system automatically generates a 3D tumor. Through systematic data integration, the anatomical location of the tumor and the relationships among abdominal organs and large blood vessels are observed in an all-around way.
(iii) 3D reconstruction of blood vessels: By selecting the marker points of the intrahepatic blood vessels, the range of blood vessels is determined. By adjusting the recognition sensitivity, the intrahepatic blood vessel information is automatically extracted to generate the portal vein, hepatic vein, and hepatic artery, which are displayed in different colors, which makes it easier to distinguish each pipeline system and accurately display the real pipeline variation in the liver. At the same time, the liver volume supplied by each branch can be calculated according to the route of each branch, which is conducive to the planning of simulated surgery. In short, the abdominal enhanced CT image is uploaded to Hisense CAS in the DICOM format to reconstruct the liver structure. The system automatically extracts the image and reconstructs the liver, gallbladder, portal vein, hepatic vein, and tumor according to the adjacent similar CT density value. The system can automatically calculate the tumor and liver volumes, carry out virtual hepatectomy, and automatically calculate the residual liver volume. According to the reconstruction results of Hisense CAS, the volume of the tumor before neoadjuvant chemotherapy and after each course of chemotherapy and the distance between the tumor and important blood vessels can be compared. At the same time, the above indices can be statistically analyzed to comprehensively evaluate the resectability of tumors.

### Surgical Methods and Postoperative Follow-Up

According to the preoperative 3D image of Hisense CAS and the virtual hepatectomy scheme, the location and size of the tumor and its relationship with surrounding organs and blood vessels are explored after routine abdominal entry. The first, second, and third hila are carefully dissected before hepatectomy. During the operation, attention is given to carefully deal with the hepatic artery, hepatic vein, short hepatic vein, and bile duct to prevent bile leakage and bleeding after the operation. When disconnecting the liver parenchyma and tumor, the liver parenchyma is cut off with a Cavitron Ultrasonic Surgical Aspirator (CUSA), and afterward, the disconnected blood vessels and bile ducts are ligated. To reduce intraoperative bleeding, the first hepatic hilum can be blocked for no more than 20 min.

The diagnosis of HB was reconfirmed after careful observation by childrens liver pathologists. The postoperative follow-up of patients in this study was completed by regular outpatient or inpatient follow-up, including abdominal imaging examination, routine blood examination, AFP determination, and liver function-related detection.

### Statistical Analysis

The measurement data are described as the mean ± standard deviation. A *t*-test was used to analyze whether there was a significant difference between the two groups. All statistical analyses in this study were carried out using the R programming language, version 3.6.0 (R Foundation), in which a *p* value* *< 0.05 was considered statistically significant.

## Results

### Patient Characteristics

A total of 21 children were included, including 13 boys and 8 girls. The average age at the time of the operation ranged from 8 months to 76 months, and the median age was 22 months. There were seventeen cases of right lobe tumors, three cases of middle lobe tumors, and one case of left lobe tumors.

### Results of the Hisense CAS 3D Reconstruction Evaluation and Analysis

**Figure 1:**
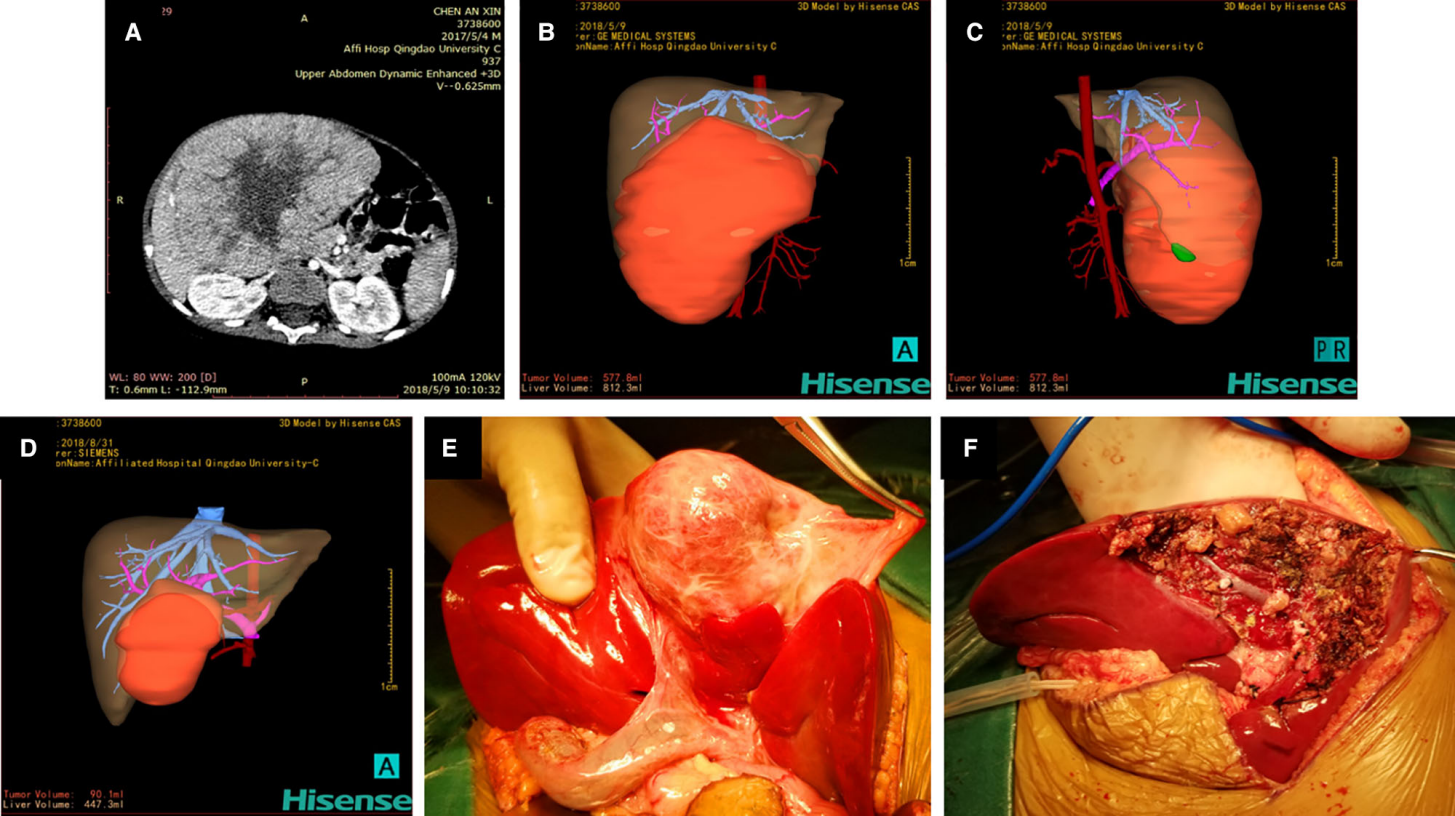
Hisense computer-assisted surgery system was used for three-dimensional reconstruction of hepatoblastoma (Case 9) at each stage. (**A**) Two-dimensional CT images of hepatoblastoma before chemotherapy. (**B**, **C**) Three-dimensional reconstruction image of hepatoblastoma before chemotherapy showing that the tumor volume and liver volume evaluated by the software are 577.8 mL and 812.3 mL respectively. (**D**) Three-dimensional reconstruction image of hepatoblastoma after three cycles of chemotherapy showing that the tumor volume is 90.1 mL and the liver volume is 447.3 mL. (**E**) Location and size of the tumor during the operation are completely consistent with the three-dimensional reconstruction image. (**F**) Intraoperative images after complete resection of the tumor.

**Figure 2:**
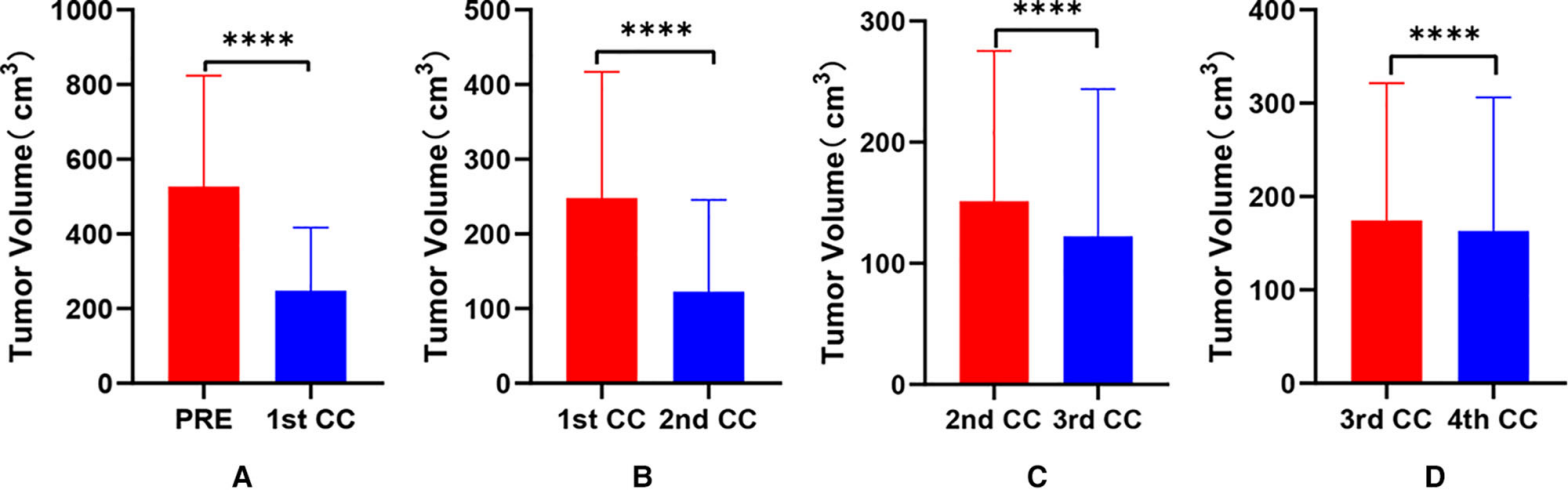
Comparative analysis of tumor volume changes in each cycle of neoadjuvant chemotherapy for hepatoblastoma. * *p* < 0.05; ** *p* < 0.01; *** *p* < 0.001; **** *p* < 0.0001.

The preoperative chemotherapy cycles of 21 children in this study were 2 patients with two cycles, 9 patients with three cycles, and 10 patients with four cycles. All cases successfully used Hisense CAS to measure and reconstruct the tumor, liver, and blood vessels around the tumor before and after chemotherapy and accurately evaluate the volume of the tumor and liver (Figure 1). The imaging changes and surgical results are shown in Table 1. The statistical results showed that the tumor diameter and tumor volume after chemotherapy were significantly lower than those before chemotherapy (Figure 2, Table 2). After chemotherapy, the distance between the tumor and portal vein and inferior vena cava increased compared with that before chemotherapy, and the difference was statistically significant. See Tables 3 and 4 for the statistical analysis results.

**Table 1:** Clinical data and results of all patients.

Case No.	PRETEXT stage		Tumor diameter (cm)		Tumor volume (cm^3^)		Distance of PV (cm)		Distance of IVC (cm)		Surgery	RSLV*R* (%)
	PRE	POS	PRE	POS	PRE	POS	PRE	POS	PRE	POS		
1	III	II	10.13	5.97	832.2	311.3	0.36	1.55	1.06	1.48	Right hemihepatectomy	59.35
2	III	III	9.59	6.27	1038.1	493.4	0.34	1.94	0.41	1.37	Right trisegmentectomy	25.49
3	III	II	9.21	5.62	579.5	192.5	0.69	3.17	1.23	1.74	Right hemihepatectomy	61.72
4	III	II	11.91	4.01	638.3	50.8	0.24	2.13	0.48	5.08	V,VI Segmentectomy	80.96
5	IV	II	12.73	4.78	1219.2	138.9	0	2.46	0.3	1.25	Right hemihepatectomy	65.32
6	III	II	6.02	3.87	542.7	104.3	0.67	1.76	1.47	2.12	Right hemihepatectomy	74.72
7	III	II	6.62	2.47	126.6	11.4	0.32	3.08	1.69	3.07	Right hemihepatectomy	68.16
8	III	II	9.35	2.83	285.2	30.0	0.47	1.46	1.28	1.58	Right hemihepatectomy	66.78
9	IV	II	11.41	5.54	577.8	90.1	0.38	1.89	1.36	3.54	Middle hepatectomy	50.48
10	III	II	10.63	2.88	518.3	46.6	0.47	2.74	0.96	2.37	Right hemihepatectomy	79.72
11	III	III	5.72	4.92	415.4	107.4	0.84	1.43	2.23	3.84	Right trisegmentectomy	26.39
12	III	III	4.68	3.75	44.5	20.3	1.53	1.94	0.63	2.36	Right trisegmentectomy	35.71
13	III	II	6.79	3.07	135.4	18.1	0.7	3.87	2.78	3.77	Middle hepatectomy	58.91
14	III	II	8.74	3.16	527.8	63.2	0.83	2.01	2.96	3.75	Right hemihepatectomy	67.51
15	III	II	10.53	3.74	537.1	64.3	0.63	1.96	1.67	3.21	Middle hepatectomy	49.85
16	IV	II	15.74	4.58	742.3	79.6	0.68	1.86	2.47	2.65	Left hepatectomy	74.79
17	III	II	10.48	4.29	605.6	74.2	0.48	1.76	1.53	1.72	Right hemihepatectomy	69.54
18	III	II	4.29	2.94	94.4	32.8	0.52	1.93	1.29	1.55	Right hemihepatectomy	71.53
19	III	II	10.45	4.17	478.3	80.7	0.59	1.78	1.42	2.58	Right hemihepatectomy	63.74
20	III	II	9.13	4.84	726.8	189.4	0.71	1.74	1.15	1.83	Right hemihepatectomy	59.83
21	III	III	5.28	4.01	395.6	94.4	0.85	1.54	1.53	1.76	Right trisegmentectomy	22.95

*Abbreviations: PRE, prechemotherapy; POS, postchemotherapy; PV, portal vein; IVC, inferior vena cava.*

**Table 2:** Changes in tumor volume in each cycle of neoadjuvant chemotherapy for HB evaluated by CAS.

Case No.	Tumor volume (cm^3^)					Preoperative chemotherapy cycle
	PRE	First chemotherapy cycle	Second chemotherapy cycle	Third chemotherapy cycle	Fourth chemotherapy cycle	
1	832.2	501.4	358.5	329.8	311.3	4
2	1038.1	716.6	534.8	510.2	493.4	4
3	579.5	351.4	227.8	211.3	192.5	4
4	638.3	207.6	75.1	50.8		3
5	1219.2	510.7	190.3	138.9		3
6	542.7	312.9	159.8	128.5	104.3	4
7	126.6	47.2	21.6	11.4		3
8	285.2	105.3	47.2	30		3
9	577.8	218.9	146.3	90.1		3
10	518.3	216.8	78.4	46.6		3
11	415.4	263.8	125.7	107.4		3
12	44.5	36.7	30.3	25.1	20.3	4
13	135.4	60.4	18.1			2
14	527.8	173.4	63.2			2
15	537.1	206.7	98.3	70.1	64.3	4
16	742.3	302.5	99.4	84.2	79.6	4
17	605.6	232.7	188.4	74.2		3
18	94.4	52.7	39.1	32.8		3
19	478.3	206.7	109.4	88.5	80.7	4
20	726.8	305.9	211.4	194.7	189.4	4
21	395.6	183.7	135.8	101.3	94.4	4

*Abbreviations: PRE, prechemotherapy.POS, postchemotherapy. PV, portal vein IVC, inferior vena cava.*

**Table 3:** Imaging comparative analysis of tumor and vascular distance evaluated by CAS in neoadjuvant chemotherapy.

	Pre-chemotherapy	Post-chemotherapy	*t*	*p*
Diameter of tumor (cm)	9.02 ± 2.91	4.17 ± 1.09	7.137	<0.05
Tumor volume (cm^3^)	526.80 ± 297.30	109.29 ± 113.02	6.015	<0.05
Distance of PV (cm)	0.59 ± 0.31	2.09 ± 0.63	9.883	<0.05
Distance of IVC (cm)	1.42 ± 0.72	2.50 ± 1.34	3.921	<0.05

*Abbreviations: PV, portal vein; IVC, inferior vena cava.*

**Table 4:** Comparative analysis of tumor volume changes in each cycle of neoadjuvant chemotherapy for hepatoblastoma.

Comparison group	Patient number	Tumor volume (cm^3^)	Tumor volume (cm^3^)	*t*	*p*
PRE VS 1st CC	21	526.7 ± 297.3	248.3 ± 168.8	7.846	<0.0001
1st CC VS 2nd CC	21	248.3 ± 168.8	122.7 ± 122.9	7.308	<0.0001
2nd CC VS 3rd CC	19	151.5 ± 124.0	122.4 ± 121.4	5.158	<0.0001
3rd CC VS 4th CC	10	174.4 ± 147.1	163.0 ± 143.3	4.866	<0.001

*Abbreviations: PRE, prechemotherapy; 1st cc, the first cycle chemotherapy; 2nd cc, the second cycle chemotherapy; 3rd cc, the third cycle chemotherapy; 4th cc, the fourth cycle chemotherapy.*

### Surgical Data and Follow-Up Results

In this study, all patients successfully had their liver tumors removed. There were twelve cases of right hemihepatectomy, four cases of right trefoil hepatectomy, one case of left lobe hepatectomy, three cases of middle lobe hepatectomy, and one case of V and VI hepatectomy. The intraoperative findings of all cases were consistent with the results of the preoperative Hisense CAS 3D reconstruction. The postoperative pathological results showed that there were seven cases of fetal type, five cases of embryonic type, three cases of mixed fetal type and embryonic type, five cases of mixed epithelial and mesenchymal type, and one case was pathologically diagnosed as small cell undifferentiated HB. All children were followed up for 5 months to 3 years. One child died of systemic metastasis 8 months after the operation. One child received a cycle of chemotherapy after the operation. Due to a serious reaction, his parents refused additional treatment and follow-up. The remaining 19 children had no complications or recurrence.

## Discussion

HB is the most common malignant liver tumor in children, but most cases cannot be operated on in one stage because of its large volume and proximity to the surrounding blood vessels (18–20). Neoadjuvant chemotherapy can significantly reduce the tumor volume so that most tumors can be completely removed and a good prognosis can be obtained (21–23). Studies have confirmed that HB can be evaluated to determine whether the tumor can be completely removed by surgery after two cycles of chemotherapy, and the judgment of its resectability mainly depends on the location and volume of the tumor after chemotherapy, the relationship with the surrounding important blood vessels, and the evaluation of the residual functional liver volume (9, 12, 14). In fact, clinical multidisciplinary consultation before HB surgery needs to consider many factors, and the formulation of the treatment plan is also extremely complex. How to evaluate the best operation opportunity, choose the best treatment scheme, and reduce the operation risk are the main problems faced by childrens tumor surgeons (24–26).

The surgical timing of complete resection of HB after chemotherapy mainly depends on the change in the tumor volume and the distance from important blood vessels. It has been reported in the literature that the tumor volume decreases significantly during neoadjuvant therapy (27). Some researchers have reported that there is no further statistically significant difference in the tumor volume after two cycles of chemotherapy (14, 28). The volume is usually calculated on the basis of 2D imaging data. There may be some errors in the measurement by different researchers. Therefore, traditional imaging is limited to the measurement of a single plane, which may not accurately reflect the overall changes in tumor size in terms of shape and volume (26).

Hisense CAS used in this study can directly reconstruct the 3D structure of liver tumors and the surrounding tissue by using 2D image data. The digital model obtained by 3D reconstruction through Hisense CAS can display the position and shape of the liver, its internal pipeline structure, and its anatomical relationship with the surrounding large blood vessels in a 3D and all-around way (29). The software can not only accurately calculate the changes in tumor volume and the distance between important blood vessels before and after chemotherapy but also accurately calculate the residual liver volume, functional liver volume, and planned resection liver volume. At the same time, it can customize the virtual surgical resection path to achieve the best preoperative planning scheme(15, 30).

The 3D reconstruction of Case 9 showed that the tumor volume before chemotherapy was 577.8 mL and the liver volume was 812.3 mL. After the fourth cycle of chemotherapy, the tumor volume was 90.1 mL, and the liver volume was 447.3 mL. It was confirmed during surgery that the tumor location was completely consistent with the Hisense CAS reconstruction image.

Our results showed that after chemotherapy, the tumor volume decreased by 52.46%–92.04%, with an average of 79.31%. The distance between the tumor and portal vein and inferior vena cava increased. In addition, our study also found that the volume of HB tumors decreased significantly after two cycles, and the tumor volume further decreased during the third and fourth cycles. Our results are different from those reported by Venkatramani et al. (12), which may be related to the different evaluation methods we used. Previous research is based on 2D CT judgment, while our research is based on 3D evaluation software, and the software directly calculates the tumor volume.

The anatomy of children’s liver is fine and complex, and the operation is difficult (31). The latest concept of precision liver surgery has the ideal goal of obtaining the best rehabilitation effect with minimum trauma and maximum liver protection (32). Therefore, precision hepatectomy seeks the best balance between radical cure of lesions and protection of the liver and reduction of body trauma (33). It is particularly important to accurately ascertain the tumor’s specific location, the scope of involvement, and adjacent relationship with surrounding blood vessels before surgery. The traditional preoperative surgical planning of pediatric liver tumors mainly depends on anatomical knowledge, B-ultrasound, CT, and other 2D imaging data, which cannot show a 3D image. At the same time, since it is limited to the surgeons personal experience and ability to read image data, it is inevitable to have a misunderstanding of conformation, which has caused challenges in actual surgical planning (26).

The Hisense CAS can automatically reconstruct the 3D morphological structure of the liver and its internal pipelines and the anatomical relationship between the tumor and its surrounding large blood vessels, which plays a great guiding role before surgery. The software has the function of automatic segmentation of the liver and lesions with artificial intelligence to realize intelligent automatic segmentation. At the same time, the software can compare and analyze the individualized 3D reconstruction data and operation planning scheme of liver tumor patients with a digital model of typical tumor cases in the equipment database, plan the operation scheme with the help of artificial intelligence technology, and provide reference guide for selecting the most accurate operation scheme (17). Comparing the reference range of the normal liver volume of children of different ages in the database and analyzing the percentage of the functional residual liver volume of the operation scheme in the normal standard liver volume of the same age can provide important help for judging the safety range of the residual liver after hepatectomy (34). The learning curve of software operation is short and does not need professional imaging technicians. Surgeons can quickly reconstruct 3D images in a short time, which is conducive to clinical popularization and use.

Computer 3D reconstruction also has some limitations. First, the technology is based on 2D image data and has certain requirements for its resolution and thin layer. Therefore, the imaging effect of Hisense CAS software is closely related to the underlying image equipment and technology. Thus, the application of this technology in economically underdeveloped areas may encounter some difficulties. Second, reconstructing the collateral circulation image of the hepatic vein from 2D and 3D images is difficult, but these collateral vessels exist widely in actual operations. Can such vessels be reconstructed before the operation? This may be the direction we need to study in the future.

## Conclusions

In conclusion, with the development of digital medicine and 3D visualization technology, pediatric liver surgery has advanced from the traditional surgery mode to a precision liver surgery mode. This study shows that Hisense CAS software can accurately evaluate the size and volume of childrens HB before and after neoadjuvant chemotherapy and the spatial relationship between the tumor and the surrounding tissues, which is helpful for the reasonable selection of an operation time. This technique makes hepatectomy in children safer and more effective and plays an important role in the diagnosis and treatment of HB.

## Data Availability

The original contributions presented in the study are included in the article/Supplementary Material, further inquiries can be directed to the corresponding author/s.
